# Editorial: Editors’ showcase—insights into molecular and cellular pathology

**DOI:** 10.3389/fcell.2023.1235103

**Published:** 2023-06-14

**Authors:** Ramani Ramchandran, Andrea Del Fattore, Rebecca Ann Wingert

**Affiliations:** ^1^ Division of Neonatology, Department of Pediatrics, Medical College of Wisconsin, Milwaukee, WI, United States; ^2^ Bone Physiopathology Research Unit, Translational Pediatrics and Clinical Genetics Research Division, Bambino Gesù Children’s Hospital, Istituti di Ricovero e Cura a Carattere Scientifico (IRCCS), Rome, Italy; ^3^ Department of Biological Sciences, University of Notre Dame, Notre Dame, IN, United States

**Keywords:** hereditary hemorrhagic telangiectasia (HHT), rhabdomyosarcoma, congenital lung disorders, cartilage formation, vasculature, bone regeneration

In this editorial, in collaboration with the Research Topic editors of the molecular and cellular pathology section in Frontiers in Cell and Developmental Biology journal, we have showcased 8 articles (https://www.frontiersin.org/research-topics/33595/editors-showcase-insights-into-molecular-and-cellular-pathology#articles) that represent the latest discoveries and advances in targets and underlying mechanisms associated with several disease pathologies. These eight articles range from specialty grand challenge (1), hypothesis and theory (1) to reviews (3) and original research (3). 64 authors worldwide contributed to this Research Topic, which has been downloaded 4,900 times and viewed by 20,000 scientists globally. These articles have already been cited 24 times in the literature, and the impact of these articles is expected to increase over time.

As a molecular and cellular pathology focused section, we receive articles on molecular targets, understanding disease etiology and new approaches to treatment for both pediatric and adult diseases. We selected these particular eight articles based on the number of views and downloads compared to articles published in the section in the same time frame. Of the eight articles, four are focused on pediatric conditions of hereditary hemorrhagic telangiectasia (HHT), pediatric soft tissue sarcoma (rhabdomyosarcoma), congenital lung disorders, and cartilage formation. The remaining articles discuss molecular targets and mechanisms underlying neural (eye), skeletal, and vascular cells.

All organs in the human body are perfused by vessels, and thus our vasculature and the cells associated with it are of prime importance to organogenesis, function, and maintenance. Endothelial cells (ECs) that line the vasculature encounter blood flow. Systemic ECs respond to flow by aligning with the direction of flow (Baeyens et al., 2016). In a specialty grant challenge article (Ramchandran), a detailed summary of the past, present, and future direction of endothelial cell biology was provided. The field of EC biology has moved from descriptive phenomenology science to one that offers deep mechanistic insight into molecular pathways and cross talk signaling between cell types in an organ. Various types of ECs within an organ are being discovered, and how these cells communicate within their environment are being delineated. Further, cellular organelles with newer function in ECs are being discovered and hypothesized. For example, cilia, a hair-like microtubule organelle found on the apical surface of ECs was hypothesized to play a role in the pathogenesis of HHT, which is characterized by defective capillary network that leads to direct connections between arteries and veins (Eisa-Beygi et al.). This defective vascular remodeling causes dilated superficial vessels referred to as artery-vein malformations (AVMs). The authors posit that loss of endothelial cilia will curtail transforming growth factor-β (TGF-β) signaling, a key signaling pathway associated with HHT pathogenesis (Rochon et al., 2016). This loss of TGF-β signaling causes vascular stability issues leading to AVMs. AVMs are proposed additions to the growing list of ciliopathies, diseases affected by dysfunctional cilia morphology or signaling. Cilia biology has benefitted greatly by studies in the retina. Yang et al. provide a comprehensive detailed review on pre-mRNA processing factors (PRPF) and their role in retinitis pigmentosa (RP), an inherited retinal disease characterized by progressive degeneration of photoreceptors and or retinal pigment epithelium leading to blindness. The review describes the mutations in PRPF genes implicated in RPs, the various animal models of disease for PRPF-RPs, and the role for these factors beyond splicing and therapeutic strategies for PRPF-RPs.

The capillary bed interfaces closely with epithelial cells to promote organ development, which is extensively studied in development of lungs. A detailed and comprehensive review that describes the cell types, molecular pathways and signals associated with embryonic and adult lung development was provided by Eenjes et al. They elegantly describe lung specification, bud formation, patterning, and outgrowth mechanisms and molecules involved in each of these process in exquisite detail. They also describe the individual cellular components in the lung and their plasticity function that can assist in regeneration mechanisms during stress or damage conditions. In the final pediatric focused article in this collection, the Research Topic highlighted is cancer-based. Rhabdomyosarcoma, a soft tissue sarcoma is classified by the presence or absence of a fusion protein (PAX3-FOXO1) into two molecular subtypes, namely, fusion positive (FP) or fusion negative (FN) tumors. FN tumors are resistant to chemotherapy and newer strategies are needed to target these cells. Perrone et al., in their original research article propose Spermine Oxidase, an enzyme that regulates polyamine catabolism, and oxidizes spermine to spermidine. These metabolites are important for apoptosis and DNA damage response in cells. Spermine oxidase enzyme is downregulated in FN tumor lines, and thus re-introducing this enzyme into FN tumor cell line is likely to induce DNA damage and sensitize these cells to irradiation. Induction of specific metabolism-related targets in cancer resistant cells warrants further analysis.

We next highlight three articles that discuss mechanisms and molecules related to bone and cartilage tissue regeneration in distinct contexts. Cartilage is thin avascular flexible tissue compared to bones which are vascularized and rigid structures containing calcified matrix. Chondrocytes are cells that form the cartilage and are involved in regeneration processes during limb development. During limb elongation in juvenile mice, the hedgehog signaling pathway receptor encoded by smoothened (*Smo*) or the regulator suppressor of fused homolog (*Sufu*) act in a chondrocyte cell autonomous manner to regulate its proliferation and differentiation. Using two different conditional deletion strategies in mice where *Smo* or *Sufu* were knocked out in different populations of chondrocytes, Xiu et al. demonstrated that in juvenile stage, hedgehog signaling functions in resting and/or columnar chondrocytes to maintain growth plate. Bone cells are composed of osteoblasts, osteocytes, osteoclasts, and progenitor cells. In the heart valve, during aging, calcification of matrix is detrimental. This process occurs via the osteogenic transition of vascular smooth muscle cells. A non-coding H19 RNA has been identified to promote osteoblastic transition of vascular smooth muscle cells by acting as a competitive endogenous RNA that sponges microRNA miR-140-5p. Xu et al. showed that H19 directly interacted with miR-140-5p, which then activated downstream signaling (Satb2-ERK/MAPK) to promote calcification. These data provide a novel mechanism for targeting arterial calcification, a common underlying condition in patients with type 2 diabetes mellitus, end-stage renal disease, obesity-related disease, and aging.

For bone regeneration, energy requirements are high, and targets that function in mitochondria, the energy producing organelles in bone and cartilage cells would be beneficial. Humanin (HN), a secreted protein that belongs to a member of mitochondrial-derived peptides acts both intracellularly and in paracrine fashion. Emerging evidence supports an important role of HN in the regulation of osteoclasts and osteoblasts, the two of the key cell types that maintain bone homeostasis. Zhu et al. provided a detailed review of HN protein in terms of molecular structure, gene expression patterns, signaling pathways and diseases influenced by this target.

These Research Topic of eight articles in this editorial showcase demonstrate the innovative approaches that these investigators have taken to address mechanistic related questions underlying disease pathology.
